# BF Integrase Genes of HIV-1 Circulating in São Paulo, Brazil, with a Recurrent Recombination Region

**DOI:** 10.1371/journal.pone.0034324

**Published:** 2012-04-02

**Authors:** Atila Iamarino, Fernando Lucas de Melo, Carla Torres Braconi, Paolo Marinho de Andrade Zanotto

**Affiliations:** Laboratory of Molecular Evolution and Bioinformatics, Department of Microbiology, Biomedical Sciences Institute-ICB II, University of São Paulo, São Paulo, Brazil; Institute of Infectious Disease and Molecular Medicine, South Africa

## Abstract

Although some studies have shown diversity in HIV integrase (*IN*) genes, none has focused particularly on the gene evolving in epidemics in the context of recombination. The *IN* gene in 157 HIV-1 integrase inhibitor-naïve patients from the São Paulo State, Brazil, were sequenced tallying 128 of subtype B (23 of which were found in non-B genomes), 17 of subtype F (8 of which were found in recombinant genomes), 11 integrases were BF recombinants, and 1 from subtype C. Crucially, we found that 4 BF recombinant viruses shared a recurrent recombination breakpoint region between positions 4900 and 4924 (relative to the HXB2) that includes 2 gRNA loops, where the RT may stutter. Since these recombinants had independent phylogenetic origin, we argue that these results suggest a possible recombination hotspot not observed so far in BF CRF in particular, or in any other HIV-1 CRF in general. Additionally, 40% of the drug-naïve and 45% of the drug-treated patients had at least 1 raltegravir (RAL) or elvitegravir (EVG) resistance-associated amino acid change, but no major resistance mutations were found, in line with other studies. Importantly, V151I was the most common minor resistance mutation among B, F, and BF *IN* genes. Most codon sites of the *IN* genes had higher rates of synonymous substitutions (*dS*) indicative of a strong negative selection. Nevertheless, several codon sites mainly in the subtype B were found under positive selection. Consequently, we observed a higher genetic diversity in the B portions of the mosaics, possibly due to the more recent introduction of subtype F on top of an ongoing subtype B epidemics and a fast spread of subtype F alleles among the B population.

## Introduction

In Brazil, BF recombinants have increasing importance in the HIV epidemic [1], [2], [3], similar to South America [4], despite subtype C [5] prevalence in the country's south region. To date, 9 BF circulating recombinant forms (CRFs) are worldwide recognized, with 6 of them sampled in Brazil (CRF28_BF, CRF29_BF, CRF38_BF, CRF39_BF, CRF40_BF, and CRF46_BF; www.hiv.lanl.gov). Also, unique recombinant forms (URFs) are commonly found [6], [7], [8]. Currently, there are 4 different antiretroviral therapies (ARV) for HIV treatment based on: (i) fusion inhibitors that inhibit conformational changes in the gp41 that are essential for fusion; (ii) reverse transcriptase (RT) inhibitors, which may be separated into 2 groups, nucleoside analogs (nucleoside reverse transcriptase inhibitors [NRTIs]) or non-nucleoside analogs (non-nucleoside reverse transcriptase inhibitors [NNRTIs]); (iii) protease (PR) inhibitors; and more recently, (iv) integrase inhibitors such as raltegravir (RAL) [9] and elvitegravir (EVG) [10]. The integrase (*IN*) gene codes for an enzyme responsible for the integration of the full proviral cDNA in the host chromosome. The HIV-1 *IN* is a 32-kDa protein with 288 amino acids (aa) expressed together with a viral PR and RT as the Pol polyprotein and released by PR cleavage during maturation. This enzyme has 3 distinct functional domains: the N-terminal domain (NTD; aa, 1–49), catalytic core domain (CCD; aa, 50–212), and C-terminal domain (CTD; aa, 213–288) [11]. The NTD has a highly conserved zinc-binding HHCC motif that stabilizes its correct folding. In the CCD, it can be found a highly conserved acidic motif, D64, D116, and E152, in all integrases and retrotransposases [11], [12]. The last domain, CTD, binds DNA nonspecifically and plays an important role in the integration process [13]. Although, the *IN* gene has become an important target in the ARV therapy, some mutations in the *IN* gene also confer resistance to the integrase inhibitors [14], [15].

Brazil started offering free access to HAART treatment in 1996 through the public health system. However, only in the beginning of 2008, RAL was approved for clinical use in patients who had HAART failure. Even though there is a large number of patients under ARV therapy, since the beginning of the epidemic, more than 217,000 people died as a consequence of AIDS, and over 630,000 might be infected by HIV [16]. The *IN* gene in Brazilian patients was previously studied by Passaes et al., 2009 [17] and Arruda et al., 2010 [18], and major resistance mutations were not found in integrase inhibitor-naïve patients, although minor mutations associated with RAL or EVG resistance were common. Nevertheless, neither of these previous works addressed the key aspect of integrase recombination. Our study aimed to sample the genetic diversity of the *IN* gene from the most prevalent subtypes in Brazil, previous to the introduction of RAL in order to evaluate its recombination profiles and drug resistance mutations in a population-genetics context, which is relevant to the epidemic in the southern cone in particular, and elsewhere in general, once the Brazilian-borne BF CRFs spread abroad [19].

## Methods

### Ethics Statements and Clinical samples

This study was submitted to and was approved by the Ethics Committee on Human Research of the Centro de Referência e Treinamento-DST/AIDS, Sao Paulo, Brazil, and all the patients signed informed consent terms. Patients were selected from those sampled and subtyped for parts of the *gag*, *pol* and *env* regions during the Viral Genetic Diversity Network (VGDN) program [20]. One hundred fifty-seven HIV-1 samples were obtained from drug-naïve and drug-treated individuals; none of which was treated with integrase inhibitors, from different cities of the São Paulo State.

### DNA Extraction, PCR, and Sequencing

DNA was extracted from the infected PBMCs by using QIAamp DNA Blood kit (Qiagen, Germany) according to the manufacturer's instructions and stored at −80°C until use. The nested-PCR and sequencing were carried out according to Van Laethem et al. (2008) [21] (Table S1). PCR products were purified using QIAquick PCR Purification Kit (Qiagen, Germany). Sequencing reactions were performed using BigDye™ Terminator version 3.0 cycle sequencing (ABI PRISM®; PE Applied Biosystems, Foster City, CA), and the products were analyzed on ABI 3100 automated DNA sequencers (PE Applied Biosystems, United States). Sequence data were edited and assembled with the CodonCode Aligner software (Gene Codes Corporation, United States). The sequences were submitted to GenBank under the accession numbers JN234023 to JN234179.

### Sequence Analysis

To expand our original dataset we included all the *IN* sequences from Brazil available in GenBank. The sequences were analyzed with the jumping profile hidden Markov model (jpHMM) program [22], [23], which uses detailed information on polymorphism of the parental populations rather than using individual parental strains and provides detailed information on the reliability of the predicted recombination breakpoints [24]. The phylogenetic analyses were performed using maximum likelihood (ML) as the optimality criterion. The ML searches were performed using GARLI v0.951 (Os×GUI) [25] under the general-time reversible (GTR) model with rates estimated from the data. Only sequences without recombination evidence within the integrase region were used for phylogenetic reconstruction. EVG and RAL resistance-associated mutations and polymorphisms were detected using the Stanford University HIV Drug Resistance Database [26].

### Positive Selection Analysis

Positive selection was determined using the single-likelihood ancestor counting (SLAC) and fixed-effects likelihood (FEL) methods implemented in the HyPhy v2.0 [27]. For the subtype B analysis, a dataset of 324 integrases (126 new sequences and 198 from GenBank); for the subtype F analysis, 80 integrases (17 new sequences and 63 references); and for subtype C analysis, 53 integrase sequences (1 new sequence and 52 references) were compiled. Only results with a *p*-value smaller than 0.05 were considered. We also compared the average rates of synonymous substitutions per site between *IN* genes estimated with SLAC from different subtypes, as an approximation of the neutral rate of change. This is justified because it is estimated at codon site positions that may change with less deleterious impact and make special sense for the *IN* genes, which are highly conserved and expected to have reduced non-synonymous rates due to purifying selection.

## Results

**Figure 1:**
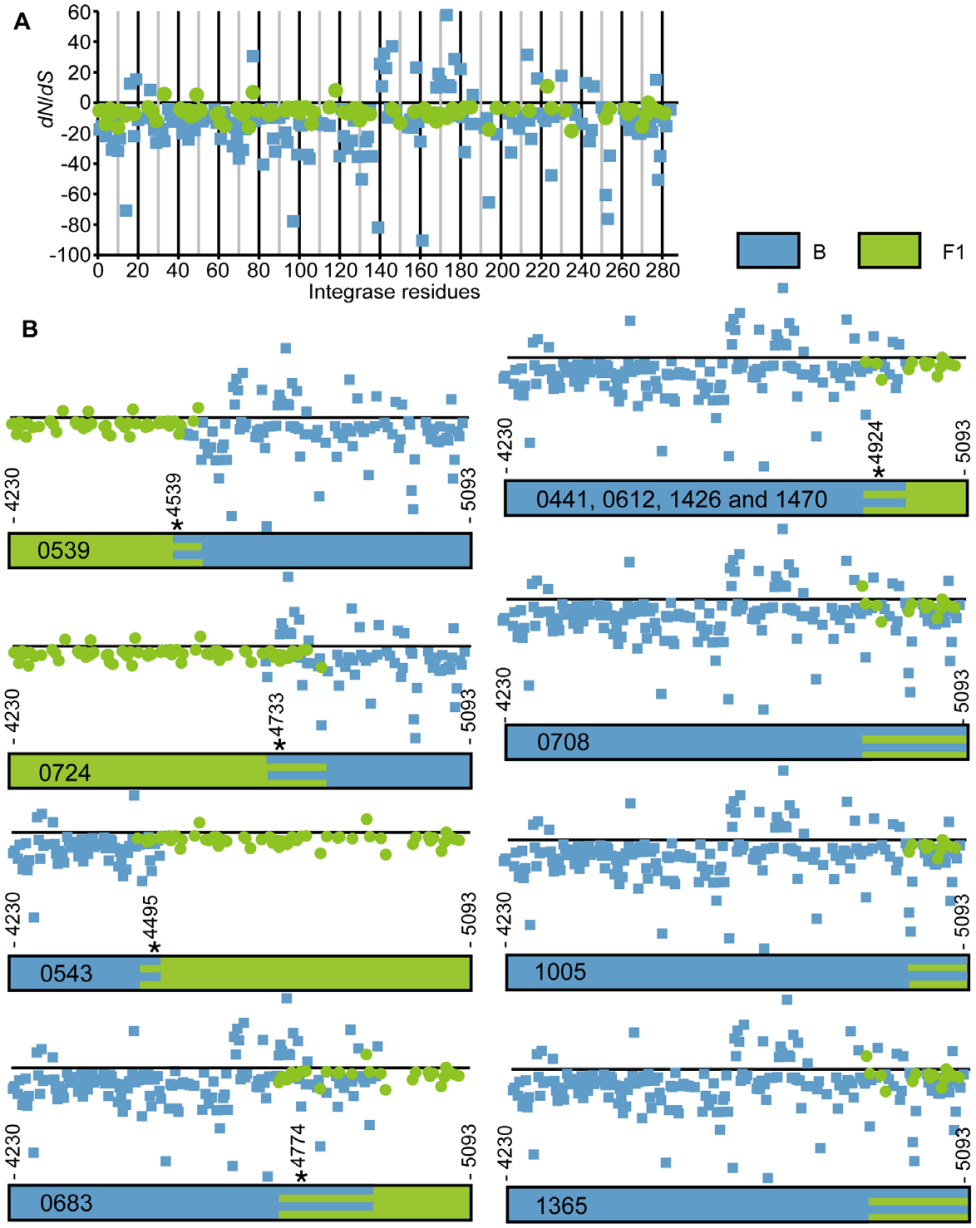
Recombination profiles and selection. Plot of the differences between non-synonymous (*dN*) and synonymous (*dS*) rates shown above in the mosaic maps of integrase (864 bp) with nucleotide sites numbered in relation to the HXB2. Rate values per codon site for the subtype B (blue squares) and subtype F (green circles). Blue and green striped portions indicate uncertainty on subtype provenance and usually include the breakpoints (indicated by an asterisk) estimated with jpHMM algorithm available online at the GOBICS server (http://jphmm.gobics.de/jphmm.html). Samples 0441, 0612, 1365, and 1470 shared the same breakpoint at nucleotide 4924 that falls inside the uncertainty region from 4899 to 4977 bp but had different recombination profiles on parts of their genomes. Sample 0708 also shared a recombination spot starting at 4899 bp, but no breakpoint could be determined.

We examined the almost complete *IN* gene (*p31*; [864 out of 867 bp] genomic positions 4230–5093 in the HXB2 reference) in 157 patients from different cities in the State of São Paulo, Brazil. The jpHMM subtyping analysis revealed that 128 integrases belonged to the subtype B, 17 to the subtype F, 11 to the BF recombinants (Figure 1), and 1 to the subtype C. To further confirm the viral subtype, we compared the integrase subtyping results with those available for other genomic regions. In addition to the recombinant viruses described above (11/157), another 31 patients were identified as carrying a BF recombinant virus, despite not having recombinant *IN* genes. Eight subtype F and 23 subtype B integrases were among the subtype BF genomes.

### The Origin of Integrase Recombinants

**Figure 2:**
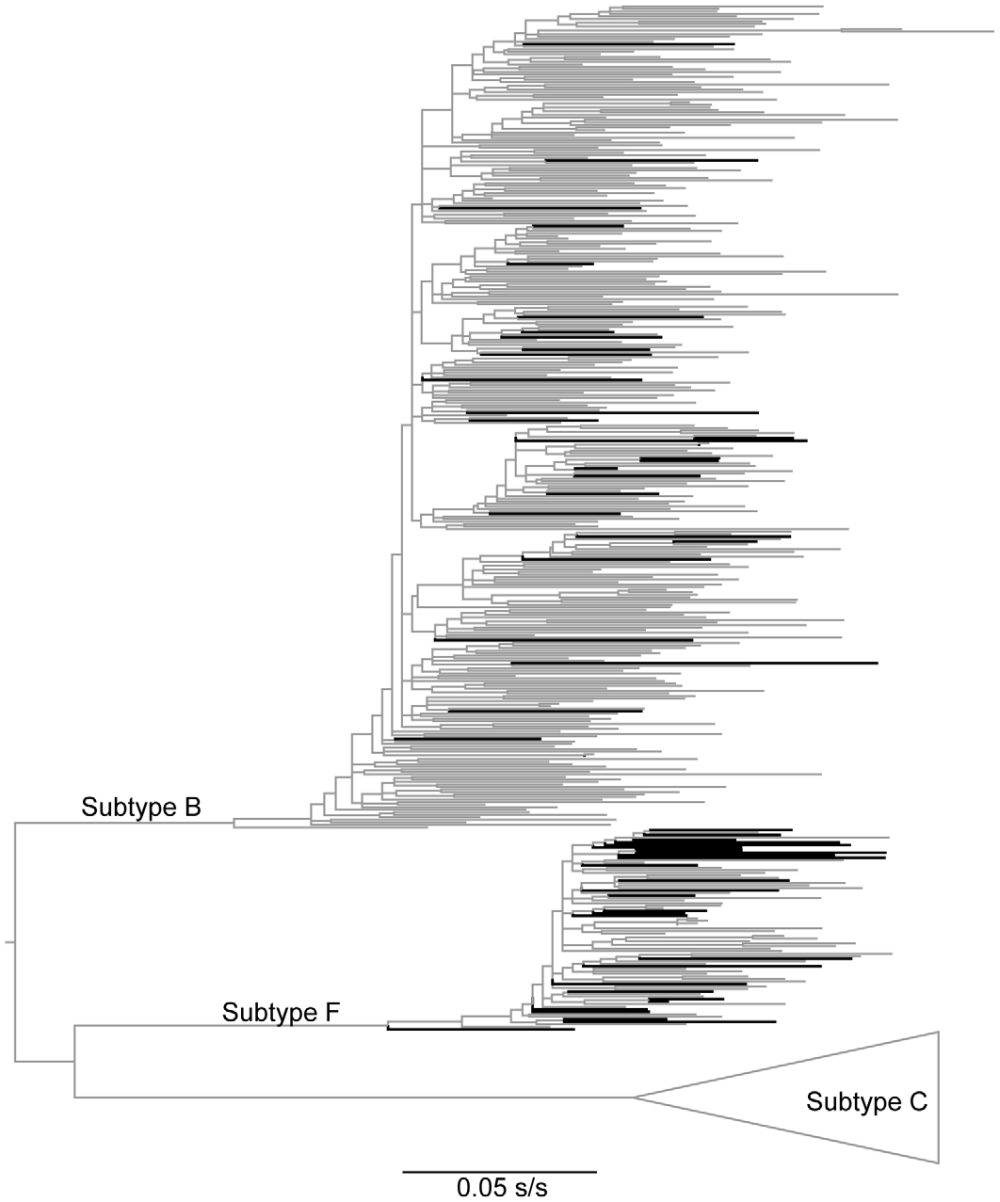
Integrase phylogeny. A maximum likelihood tree for 459 non-recombinant integrase sequences from Brazil (145 generated by us and 314 from other studies) inferred with GARLI v0.95. Thick branches indicate lineages known to be recombinants on other regions of their genomes. The tree was midpoint rooted, which agrees precisely with the known relationship among HIV-1 group M subtypes [34]. Subtype C sequences are collapsed for clarity. This tree shows a large number of potential independent origination events of BF recombinants.

Since our results indicated that the vast majority of BF recombinant viruses were not monophyletic (Figure 2), we needed to consider if they had distinct, independent origins as if emerging from distinct co-infection events, despite similarities in their inferred breakpoints. Actually, the high number of URFs, seen in Figure 2, agreed with the reports on a large number of unique BF forms in the Brazilian HIV-1 epidemic [3], [6], [7]. However, 4 samples shared a recombination breakpoint region between positions 4900 and 4924 (e.g., 0441, 0612, 1426, and 1470) (Figure 1), which could be explained by a shared evolutionary history or maybe by the presence of a recombination hotspot in that particular position in the genome, among other possibilities. To investigate if these recombinants have a common origin, we did a phylogenetic analysis on the first 500 nt (recombination-free segments), including all the subtype B integrases and 8 recombinant integrases (0441, 0612, 0683, 0708, 1005, 1365, 1426, and 1470) that shared this 500-bp region with the subtype B.

**Figure 3:**
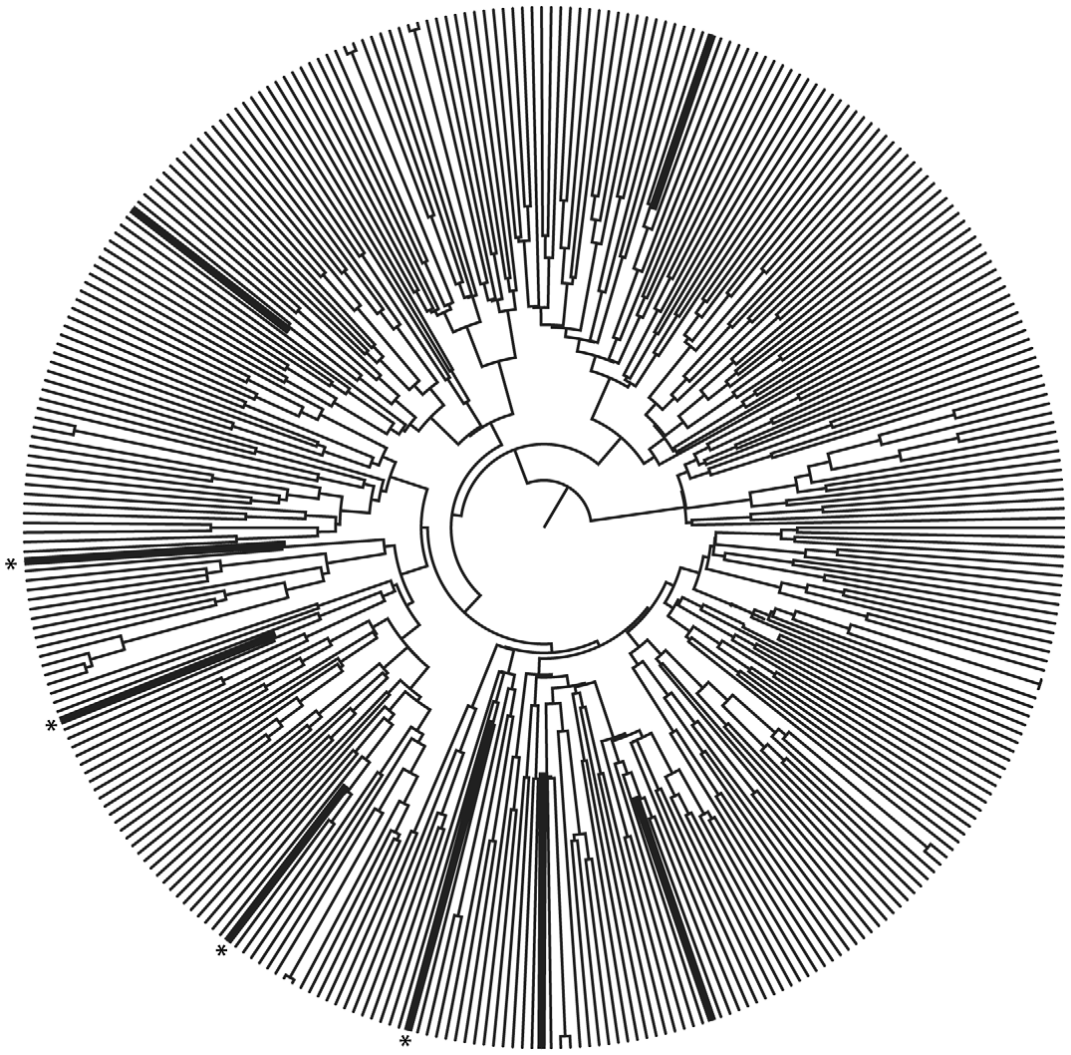
Phylogenetic tree using recombination-free segment. The cladogram summarizes the maximum clade credibility (MCC) tree including all subtype B integrases and the recombinant integrase sequences sharing the 500 bp subtype B segment. It shows reconstructions in “recombinant” and “non-recombinant” state transitions on a set of 5000 plausible trees by using the symmetric CTMC model for discrete character reconstruction with BEAST. Recombinant sequences are shown as thick branches, while asterisks at the tips of the MCC tree show sequences that share a breakpoint at position 4924. Branch lengths only help the visualization of transition events and are not proportional to either quantity of events or substitution per sites.

Nevertheless, only four of the BF integrase sequences had well defined breakpoints, but a short F portion of around 150 bp-long, making them unsuitable for robust phylogenetic inference on the origin of the F fragments alone. Figure 3 shows that recombinant sequences dispersed all over the tree as if they had independent origins. We found the posterior probability of a possible shared ancestral node for all the recombinants (*i.e.*, testing for monophyly) to be zero on a set of 5000 plausible trees sampled at stationarity during 2 independent Markov chain Monte Carlo (MCMC) runs, each consisting of 20 million steps with the BEAST v1.6.1. Moreover, we also reconstructed the “recombinant” and “non-recombinant” states on the same set of plausible trees by using the symmetric CTMC model for discrete character reconstruction with BEAST and found that all recombinant states had posterior probabilities of 0.99 for independent events of transition from “non-recombinant” to “recombinant” nested within the clades of “non-recombinant” integrases with a posterior probability of 1 (Figure 3). We did not find any other HIV CRF genome with a recombination breaking point at that position. The recurrence of independently acquired breaking points suggests some sort of underlying facilitating process at the molecular level. Crucially, the region where recombination breakpoints had the highest posterior probabilities converged in 2 loops, comprising a highly structured viral genomic RNA (gRNA) region [28] (Figure S1) that is prone to RT stalls and strand transfer during HIV cDNA synthesis involved in HIV recombination [28].

### Relevant Integrase Polymorphisms

One hundred and thirty-two of the 157 patients (84.08%) had already been treated with at least 1 drug regimen (mean, 3; up to 13 distinct drug combinations), 85.6% of which were subjected to HAART. However, none of them received integrase inhibitors. Forty percent of the drug-naïve and 45.5% of the drug-treated patients had at least 1 RAL or EVG resistance-associated amino acid change, which were values lower than those recently found in the State of São Paulo [18]. No major resistance mutations (66I, 92Q, 140A/S, 143R/C, 147G, 148H/R/K, and 155H) were found. Half of the 16 subtype F integrases had minor resistance-associated mutations. V151I was the most frequent and found to be present in 65.2% (5/8) of the cases. The polymorphisms E138D, M154I, and S230N were also found in 1 sample each. Our single subtype C patient was drug-naïve and had no resistance-associated mutation. Likewise, V151I (25%) was the most common minor resistance mutation among the BF recombinant integrases, which also had 1 sample sporting M154I. In the subtype B samples, V151I was also the most frequent change in 23.3% of the patients. Interestingly, unlike the F and BF samples, S230N was the second most common mutation, present in 8.5% of the subtype B sequences. Other B resistance-associated mutations were L74M (0.8%), T97A (1.6%), E138K (0.8%), M154I (3.9%), M154L (0.8%), E157K (2.3%), G163R (3.1%), and I203M (3.1%). The complete list of polymorphisms is available in Table S2.

### Selection Regimen

Figure 1 shows the difference between non-synonymous (*dN*) and synonymous (*dS*) rates at each codon of the *IN* genes. Positive values indicate an excess of *dN* that is indicative of a directional or positive selection, while negative values indicate codons under higher *dS* rates, suggestive of purifying or negative selection. The extent of the differences indicates the strength of selection. Based only on *dS* values, it can be observed higher genetic diversity in the B portions of the mosaics, which is in agreement with the notion of a more recent introduction of the F on top of a B epidemic, possibly in concert with a fast spread of F alleles among the B population. The fact that most of the codon sites of the integrase had higher *dS* agrees with its functional relevance for viral replication revealed by the strong negative selection on deleterious changes. Nevertheless, several codon sites mainly in the subtype B were found under positive selection using the SLAC method, despite the fact that the integrase is relatively conserved compared to other HIV-1 genes. Some of the positively selected sites, such as residue 72 in the subtype B and F integrases and residues 151 and 201 in the subtype B, were further confirmed using FEL. Moreover, the subtype F integrases had 3 additional positively selected sites at the CCD. Subtype B, on the other hand, presented many positively selected sites, including residues with important biological functions, such as the active site loop (aa, 140) [11]. Interaction with human lens epithelium-derived growth factor (LEDGF/p75; aa: 161, 166, 168, and 171) [29], [30], DNA binding (aa, 163) [30], and the nuclear import signal (aa, 165 and 166) [31]. At the CTD, positive selection was found only in the subtype B integrase, including at the DNA binding sites (aa: 230, 234, and 284) [29]. At the NTD, the subtype B and subtype F had 2 positively selected sites (at residues: 17 and 24 and at residues 31 and 45, respectively). Furthermore, the most common BF integrase recombination profile (breakpoint at residue 232) preserved the positively selected NTD and CCD domains, and the subtype F conserved the DNA binding site at CTD. We also found that the B integrases (*dS_B_* = 14.42±1.88) had a 4.74 times higher rate of change estimated at silent sites (*dS*) compared to F integrases (*dS_F_* = 3.04±0.44) (*dS_B_*>*dS_F_*, with a probability of 5.9E-28 of being the same value given a Student's paired *t*-test, with a 2-tailed distribution). This huge difference entails a larger population size and possibly an earlier entry of the subtype B in our epidemics. The complete list of positively selected sites is available in Table S3.

## Discussion

We found that the *IN* gene of the HIV-1 circulating in the epidemics in the São Paulo State displayed a diverse plethora of BF recombinants. Our findings are relevant since not much is known about the diversity of circulating viruses and their background resistance-associated polymorphisms in general. The absence of major integrase resistance mutations in our integrase inhibitor-naïve patients, 79% of which were under ARV drugs such as RT and PR inhibitors, is in line with other studies [17], [18] and justifies the use of this therapeutic strategy. None of the 11 recombinant integrases we found shared the same breakpoints with the already described BF CRFs from Brazil, although CRF47_BF from Spain has a breakpoint close to those found in 0441, 0612, 1426, and 1470. These results show the importance of sequencing different genomic regions for improving HIV subtyping and give information on the potential of the underestimation of the real diversity of BF recombinants in our epidemics. Moreover, we tested the hypothesis that the recombinant genes originated from a single common ancestral recombination event (testing for monophyly) by calculating the posterior probability (that was zero) of monophyly on a set of 5000 plausible trees sampled at stationarity during 2 independent MCMC runs. We also checked these results by reconstructing the “recombinant” and “non-recombinant” states on the same set of plausible trees. This exercise also reinforced (with posterior probability of 1) the hypothesis of an independent origin of “recombinant” from “non-recombinant” states within the clades of “non-recombinant” integrases. Critically, both independent approaches provided strong statistical support for the notion that the recombinant sequences we found originated from distinct recombination events. That being the case and given the fact that the recurrent recombination region (Figure S2) fell within a secondary gRNA structural feature known to propitiate recombination, we argue that it may indicate a potential recombination hotspot, which was not previously reported for HIV-1 in general and BF recombinants in particular. Nevertheless, *IN* is a very conserved region and therefore prone to recombination [32], [33]. Moreover, we cannot be sure of the extent to which our estimates could have been influenced by the recombination itself. This is because recombinants with common origin could differentiate later by distinct subsequent recombination events with divergent parental strains (i.e., second-generation recombinants). We also observed a 4.7-times higher accumulation of diversity in the B subtypes compared to F subtypes in the *IN* genes. Since there was a reduction in the number of positively selected sites in the F regions and given that the most positively selected sites were detected at the B portion of recombinant forms, we argue that this higher genetic diversity could be explained by a more recent introduction of F on top of a B epidemics and a fast spread of F alleles among the larger B population. It remains to be investigated if the advantage conferred by the F portions may have to do with the intrinsic F integrase functionality or adaptive changes acquired following coinfection.

## Supporting Information

Figure S1 **Breakpoint region in secondary RNA structure.** Secondary structure of the HIV genomic RNA (gRNA) numbered according to HXB2. The red region marks the recombination hotspot from bases 4900 to 4924. Adapted from Watts J. M. et al. (2009).
(TIF)

Figure S2 **Recombination posterior probabilities.** Posterior probabilities according to jpHMM of the subtypes for patients 0441 (A), 0612 (B), 1426 (C), and 1470 (D).
(TIF)

Table S1 Primers used for nested amplification of the HIV-1 IN region from according to Van Laethem K.V. et al. (2008).
(DOC)

Table S2 Complete list of polymorphisms found at integrase sequences. Minor resistance mutations are shown in red.
(DOC)

Table S3 Positively selected sites in the HIV-1 integrase gene coding region.
(DOC)
