# Supplementary figures and images for: BF Integrase Genes of HIV-1 Circulating in São Paulo, Brazil, with a Recurrent Recombination Region
Source: PLoS One. 2012 Apr 2;7(4):e34324. doi: 10.1371/journal.pone.0034324 (PMC3317518; doi:10.1371/journal.pone.0034324)

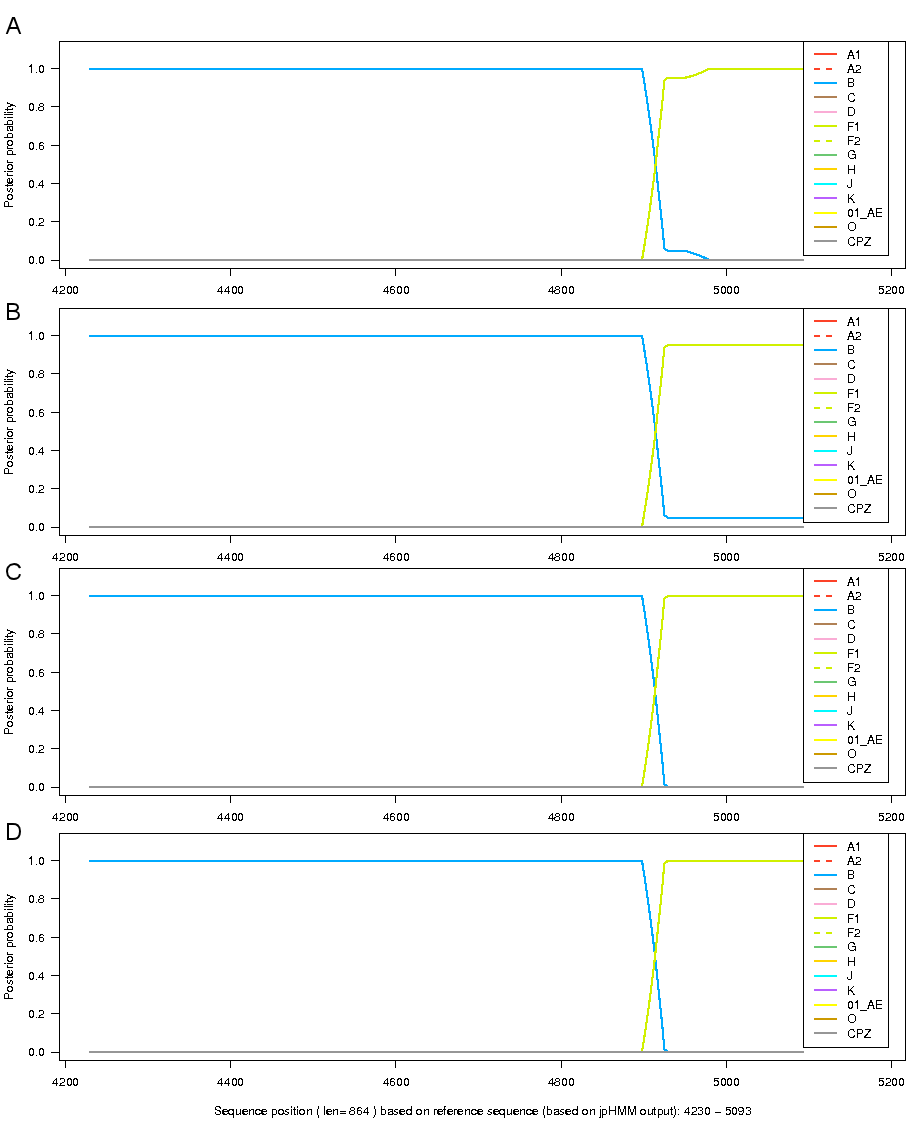

Supplement: Figure S2 — Recombination posterior probabilities. Posterior probabilities according to jpHMM of the subtypes for patients 0441 (A), 0612 (B), 1426 (C), and 1470 (D). (TIF) [file pone.0034324.s002.tif]
