# Supplementary material for: BF Integrase Genes of HIV-1 Circulating in São Paulo, Brazil, with a Recurrent Recombination Region
Source: PLoS One. 2012 Apr 2;7(4):e34324. doi: 10.1371/journal.pone.0034324 (PMC3317518; doi:10.1371/journal.pone.0034324)
Supplement: Table S1 — Primers used for nested amplification of the HIV-1 IN region from according to Van Laethem K.V. et al. (2008). (DOC) [file pone.0034324.s003.doc]

**Table S1.**  Primers used for nested ampliﬁcation of the HIV-1 IN region from according to Van Laethem K.V. et al. (2008).

| **Primers** | **Positions (HXB2)** | **Sequence (5’→3’)** |
| --- | --- | --- |
| **1^st^ round** |  |  |
| KVL068 (+) | 3854–3880 | AGGAGCAGAAACTTWCTATGTAGATGG |
| KVL069 (-) | 5956–5982 | TTCTTCCTGCCATAGGARATGCCTAAG |
| **2^nd^ round** |  |  |
| KVL070 (+) | 4013–4041 | TTCRGGATYAGAAGTAAAYATAGTAACAG |
| KVL084 (-) | 5243–5265 | TCCTGTATGCARACCCCAATATG |
