# Supplementary material for: BF Integrase Genes of HIV-1 Circulating in São Paulo, Brazil, with a Recurrent Recombination Region
Source: PLoS One. 2012 Apr 2;7(4):e34324. doi: 10.1371/journal.pone.0034324 (PMC3317518; doi:10.1371/journal.pone.0034324)
Supplement: Table S2 — Complete list of polymorphisms found at integrase sequences. Minor resistance mutations are shown in red. (DOC) [file pone.0034324.s004.doc]

**Table S2.**  Complete list of polymorphisms found at integrase sequences. Minor resistance mutations are shown in red.

| **Subtype B** | **I72V** (50,39%), **V31I** (37.98%), **V201I** (37.98%), **T125A** (31.78%), **L101I** (29.46%), **S17N** (24.03%), **V151I** (23.3%), **T206S** (21.71%), **E11D** (19.38%), **L28I** (18.6%), **L234I** (17.83%), **A21S** (17.05%), **S39C** (17.05%), **T124N** (17.05%), **D256E** (16.28%), **S119P** (14.73%), **T122I** (14.73%), **I113V** (13.18%), **S283G** (13.18%), **K7E** (12.4%), **E10D** (12.4%), **V32I** (11.63%), **T124A** (11.63%), **K156N** (10.08%), **D25E** (8.53%), **M50I** (8.53%), **S230N** (8.53%), **D253E** (8.53%), **V126L** (7.75%), **K215N** (7.75%), **T218I** (7.75%), **K14R** (6.98%), **I220L** (6.98%), **A265V** (6.98%), **D6T** (6.2%), **V37I** (6.2%), **S119T** (6.2%), **V165I** (6.2%), **Q216H** (6.2%), **D6N** (5.43%), **A23V** (5.43%), **I135V** (5.43%), **T218S** (5.43%), **D232E** (5.43%), **D6S** (4.65%), **S119G** (4.65%), **A205S** (4.65%), **D6E** (3.88%), **S24G** (3.88%), **K34R** (3.88%), **M50T** (3.88%), **T112A** (3.88%), **T125V** (3.88%), **M154I** (3.88%), **N222K** (3.88%), **R284G** (3.88%), **K7R** (3.1%), **K7Q** (3.1%), **R20K** (3.1%), **D41N** (3.1%), **L45V** (3.1%), **K111R** (3.1%), **G163E** (3.1%), **G163R** (3.1%), **K188R** (3.1%), **I203M** (3.1%), **K219N** (3.1%), **Y227F** (3.1%), **D279G** (3.1%), **E13D** (2.33%), **M22L** (2.33%), **S24N** (2.33%), **P30A** (2.33%), **V88I** (2.33%), **T112I** (2.33%), **E157K** (2.33%), **R187K** (2.33%), **G193E** (2.33%), **I220V** (2.33%), **N222H** (2.33%), **L234H** (2.33%), **R269K** (2.33%), **D278N** (2.33%), **D286N** (2.33%), **G4R** (1.55%), **L45I** (1.55%), **L45Q** (1.55%), **G59R** (1.55%), **I60M** (1.55%), **I73V** (1.55%), **G82R** (1.55%), **I84M** (1.55%), **T97A** (1.6%), **V126M** (1.55%), **D167E** (1.55%), **H171Q** (1.55%), **K173R** (1.55%), **F181L** (1.55%), **D207E** (1.55%), **K219Q** (1.55%), **L234V** (1.55%), **L234F** (1.55%), **N254K** (1.55%), **I268L** (1.55%), **V281M** (1.55%), **S283N** (1.55%), **G4E** (0.78%), **Q9P** (0.78%), **E13K** (0.78%), **E13Q** (0.78%), **S17T** (0.78%), **S17C** (0.78%), **W19*** (0.78%), **S24A** (0.78%), **N27G** (0.78%), **P30Q** (0.78%), **P30L** (0.78%), **E35K** (0.78%), **E35A** (0.78%), **E35Q** (0.78%), **I36L** (0.78%), **S39CS** (0.78%), **S39N** (0.78%), **Q44H** (0.78%), **L45P** (0.78%), **K46Q** (0.78%), **G47E** (0.78%), **A49P** (0.78%), **M50L** (0.78%), **G52R** (0.78%), **V54I** (0.78%), **S57N** (0.78%), **I60V** (0.78%), **W61*** (0.78%), **L63V** (0.78%), **E69K** (0.78%), **E69D** (0.78%), **G70D** (0.78%), **G70E** (0.78%), **K71R** (0.78%), **I72T** (0.78%), **L74I** (0.78%), **L74M** (0.78%), **V75A** (0.78%), **V77A** (0.78%), **A80S** (0.78%), **I84L** (0.78%), **E87D** (0.78%), **P90S** (0.78%), **A91T** (0.78%), **A91S** (0.78%), **E96D** (0.78%), **Y99F** (0.78%), **G106A** (0.78%), **K111Q** (0.78%), **K111N** (0.78%), **K111A** (0.78%), **T112IT** (0.78%), **D116N** (0.78%), **S119R** (0.78%), **T122P** (0.78%), **T124S** (0.78%), **T125AT** (0.78%), **K127R** (0.78%), **W131*** (0.78%), **W132*** (0.78%), **G134E** (0.78%), **G134K** (0.78%), **K136Q** (0.78%), **K136N** (0.78%), **K136R** (0.78%), **E138K** (0.78%), **F139S** (0.78%), **E152K** (0.78%), **M154L** (0.78%), **K156S** (0.78%), **G163V** (0.78%), **G163R** (0.78%), **R166K** (0.78%), **E170K** (0.78%), **K173E** (0.78%), **V176E** (0.78%), **F181Y** (0.78%), **H183Y** (0.78%), **G190E** (0.78%), **G192E** (0.78%), **G193R** (0.78%), **S195T** (0.78%), **E198Q** (0.78%), **E198K** (0.78%), **E198D** (0.78%), **R199K** (0.78%), **V201IV** (0.78%), **I204V** (0.78%), **T206A** (0.78%), **D207N** (0.78%), **I208M** (0.78%), **E212T** (0.78%), **Q216K** (0.78%), **Q216N** (0.78%), **I217V** (0.78%), **K219T** (0.78%), **I220F** (0.78%), **I220M** (0.78%), **Q221H** (0.78%), **R224Q** (0.78%), **D229E** (0.78%), **S230G** (0.78%), **S230H** (0.78%), **D232N** (0.78%), **K240R** (0.78%), **K244E** (0.78%), **D253Y** (0.78%), **N254Q** (0.78%), **S255G** (0.78%), **D256K** (0.78%), **I257T** (0.78%), **V259I** (0.78%), **R262K** (0.78%), **K264R** (0.78%), **I267V** (0.78%), **D270N** (0.78%), **D270E** (0.78%), **D270H** (0.78%), **G272R** (0.78%), **K273R** (0.78%), **A282T** (0.78%), **S283D** (0.78%) |
| --- | --- |
| **Subtype BF** | **S283G** (83.33%), **V201I** (75%), **I72V** (66.67%), **L101I** (66.67%), **D256E** (66.67%), **L234V** (58.33%), **E11D** (50%), **S17N** (41.67%), **T125A** (41.67%), **V31I** (33.33%), **I84L** (25%), **S119P** (25%), **S119T** (25%), **T124A** (25%), **V151I** (25%), **L28I** (16.67%), **V32I** (16.67%), **V37I** (16.67%), **I113V** (16.67%), **T122I** (16.67%), **K156N** (16.67%), **V165I** (16.67%), **D6E** (8.33%), **E10A** (8.33%), **E10D** (8.33%), **S17T** (8.33%), **N18S** (8.33%), **S24G** (8.33%), **M50L** (8.33%), **G59E** (8.33%), **I60M** (8.33%), **I72T** (8.33%), **L74I** (8.33%), **G82R** (8.33%), **I84M** (8.33%), **E96D** (8.33%), **F100Y** (8.33%), **K103R** (8.33%), **T112A** (8.33%), **T112V** (8.33%), **N117K** (8.33%), **S119A** (8.33%), **T124N** (8.33%), **T125V** (8.33%), **G134R** (8.33%), **I135V** (8.33%), **K136Q** (8.33%), **K136L** (8.33%), **K136N** (8.33%), **V150A** (8.33%), **G193R** (8.33%), **A205S** (8.33%), **T206S** (8.33%), **K215Q** (8.33%), **K215N** (8.33%), **T218I** (8.33%), **L234I** (8.33%), **N254H** (8.33%), **A265V** (8.33%), **R269K** (8.33%) |
| **Subtype F** | **T124A** (87.5%), **K136Q** (87.5%), **V201I** (87.5%), **D256E** (87.5%), **S283G** (81.25%), **A205S** (75%), **L234V** (75%), **L101I** (68.75%), **S17N** (62.5%), **T125A** (62.5%), **I72V** (56.25%), **I84L** (56.25%), **S119T** (56.25%), **T218I** (56.25%), **V165I** (43.75%), **V151I** (40%), **E11D** (37.5%), **V31I** (25%), **S119P** (25%), **E10D** (18.75%), **R20K** (18.75%), **A21S** (18.75%), **V32I** (18.75%), **V54I** (18.75%), **T112I** (12.5%), **I135V** (12.5%), **G163S** (12.5%), **L234I** (12.5%), **K7R** (6.25%), **E13D** (6.25%), **K14R** (6.25%), **W19*** (6.25%), **S24N** (6.25%), **L28I** (6.25%), **V37I** (6.25%), **S39N** (6.25%), **S39C** (6.25%), **D41N** (6.25%), **M50L** (6.25%), **M50I** (6.25%), **V54T** (6.25%), **D55N** (6.25%), **I60V** (6.25%), **K103R** (6.25%), **K111R** (6.25%), **T112V** (6.25%), **I113V** (6.25%), **D116N** (6.25%), **S119A** (6.25%), **G134E** (6.25%), **E138D** (6.25%), **F139Y** (6.25%), **M154I** (6.25%), **I161T** (6.25%), **G163N** (6.25%), **R199K** (6.25%), **T218S** (6.25%), **Y227F** (6.25%), **S230N** (6.25%), **D232E** (6.25%), **R269K** (6.25%) |
