# Supplementary material for: BF Integrase Genes of HIV-1 Circulating in São Paulo, Brazil, with a Recurrent Recombination Region
Source: PLoS One. 2012 Apr 2;7(4):e34324. doi: 10.1371/journal.pone.0034324 (PMC3317518; doi:10.1371/journal.pone.0034324)
Supplement: Table S3 — Positively selected sites in the HIV-1 integrase gene coding region. (DOC) [file pone.0034324.s005.doc]

**Table S3.**  Positively selected sites in the HIV-1 integrase gene coding region.

|  | N-terminal Domain | | Catalytic Core Domain | | | | | | | | | | | | | | | | | | | C-terminal Domain | | | | | |
| --- | --- | --- | --- | --- | --- | --- | --- | --- | --- | --- | --- | --- | --- | --- | --- | --- | --- | --- | --- | --- | --- | --- | --- | --- | --- | --- | --- |
| **AA** | **17** | **24** | **72** | **134** | **135** | **136** | **137** | **140Δ** | **151** | **159*** | **160** | **161L** | **162** | **163** | **164** | **165†** | **166L †** | **168L** | **171L** | **201** | **206** | **218** | **230*** | **234*** | **265*** | **283** | **284** |
| Subtype B | 15.29 | 8.52 | 30.44 | 25.57 | 10.88 | 32.20 | 22.78 | 37.00 | 23.14 | 10.04 | 19.01 | 13.72 | 11.40 | 10.76 | 57.71 | 11.43 | 10.28 | 28.60 | 22.10 | 31.54 | 15.88 | 17.70 | 12.96 | 10.87 | 15.06 | 12.51 | 5.00 |
| p-value | 0.01 | 0.02 | 0.00 | 0.00 | 0.01 | 0.00 | 0.00 | 0.00 | 0.00 | 0.00 | 0.00 | 0.00 | 0.04 | 0.02 | 0.00 | 0.00 | 0.00 | 0.00 | 0.00 | 0.00 | 0.00 | 0.00 | 0.00 | 0.00 | 0.00 | 0.00 | 0.03 |
|  |  |  |  |  |  |  |  |  |  |  |  |  |  |  |  |  |  |  |  |  |  |  |  |  |  |  |  |
|  |  |  |  |  |  |  |  | L | Residues involved in interaction with human lens epithelium-derived growth factor (LEDGF/p75) | | | | | | | | | | | | | |  |  |  |  |  |
|  | N-terminal Domain | | Catalytic Core Domain | | |  |  | * | DNA Binding sites | | | |  |  |  |  |  |  |  |  |  |  |  |  |  |  |  |
| **AA** | **31** | **45** | **72** | **112** | **211*** |  |  | Δ | Active Site Loop | | | | | | | | | | |  |  |  |  |  |  |  |  |
| Subtype F | 5.68 | 5.13 | 6.83 | 8.12 | 10.89 |  |  | † | Nuclear importation signal | | | | | | | | | | | | |  |  |  |  |  |  |
| p-value | 0.03 | 0.03 | 0.02 | 0.00 | 0.00 |  |  | _ | Conserved sites | | | | | | | | | |  |  |  |  |  |  |  |  |  |
